# Comparison of intracerebrospinal fluid methotrexate by Ommaya reservoir versus lumbar puncture in leptomeningeal carcinomatosis with hydrocephalus: a retrospective cohort study

**DOI:** 10.3389/fneur.2026.1848001

**Published:** 2026-07-28

**Authors:** Kun Hong, Chang Liu, Yu Zhang, Yi Li, Qing Li, Junying He, Hui Bu

**Affiliations:** 1 Department of Neurology, The Second Hospital of Hebei Medical University, Shijiazhuang, Hebei, China; 2 Institute of Cardiocerebrovascular Disease, Shijiazhuang, Hebei, China; 3 Neurological Laboratory of Hebei Province, Shijiazhuang, Hebei, China

**Keywords:** hydrocephalus, intracerebrospinal fluid chemotherapy, leptomeningeal carcinomatosis, lumbar puncture, Ommaya reservoir

## Abstract

**Background:**

Leptomeningeal carcinomatosis (LC) complicated by hydrocephalus carries a poor prognosis. Intracerebrospinal fluid (intra-CSF) chemotherapy is a main treatment, but the optimal route of administration remains unclear.

**Methods:**

This retrospective cohort study included 36 LC patients with hydrocephalus who received intra-CSF methotrexate via Ommaya reservoir (OR, *n* = 15) or lumbar puncture (LP, *n* = 21). Efficacy, safety, and overall survival (OS) were compared. Prognostic factors were analyzed using Cox regression.

**Results:**

The OR group achieved significantly higher overall response rate (86.7% vs. 47.6%, *p* = 0.016) and clinical response rate (100% vs. 52.4%, *p* = 0.002), alongside superior intracranial pressure relief (*p* = 0.042). Rates of complications and chemotherapy-related toxicities were comparable in both arms. Median overall survival (OS) was 54.7 weeks for OR patients versus 44.1 weeks for LP patients, without statistically significant intergroup difference (*p* = 0.53). After adjusting for primary tumor subtypes in multivariate Cox regression, extraneural metastasis remained an independent adverse prognostic factor (HR = 4.251, *p* = 0.009), whereas targeted therapy independently predicted prolonged OS (HR = 0.101, *p* < 0.001). Treatment response lost independent predictive significance after full adjustment (*p* = 0.055). Tumor subtype showed no significant correlation with survival outcomes.

**Conclusion:**

In LC patients with hydrocephalus, intra-CSF methotrexate via OR does not prolong OS but yields superior symptom relief and intracranial pressure control without increased adverse events. Concurrent targeted therapy is independently linked to favorable long-term survival; extraneural metastasis confers higher mortality risk.

## Background

Leptomeningeal carcinomatosis (LC) is defined as multifocal or diffuse metastases of leptomeninges from systemic tumors, with or without associated lesions of brain parenchyma (1, 2). An increased incidence of LC has occurred in part due to the development of effective antineoplastic treatments and improvement of diagnostic tools. Common neoplastic etiologies include breast, lung, gastro-intestinal, melanoma, and lymphoma (3, 4). The LC-related clinical manifestations and neuroimaging findings are variable and nonspecific as the hallmark multifocal involvement. To date, the diagnosis of LC is still challenging. The diagnostic evaluation is on clinical findings, cerebrospinal fluid (CSF) analysis, and neuroimaging features (5). Currently, there are no standard treatment guidelines for LC. Although treatments have traditionally included systemic chemotherapy, intrathecal (IT) chemotherapy, whole brain or focal radiotherapy, immunotherapy, and supportive care, the therapeutic goal remains palliation, with most patients surviving four to 6 months with effective combined modality treatments (6, 7).

It has been found that, over one-quarter of patients with LC may develop into symptomatic hydrocephalus (8), which is positive correlation with worse symptoms, including headache, nausea, vomiting, visual disturbances, seizures, nerve palsies and unconsciousness (9). LC can present with both communicating and obstructive hydrocephalus. The former is caused by increased CSF protein, which obstructs CSF absorption through the arachnoid granulations (8), while the latter is due to the obstruction of the CSF pathway from large parenchymal or intraventricular mass, or posterior fossa LC (10, 11). Yet, there exists no standard therapy for its management. So far, there is some clinical evidence that intracerebrospinal fluid (intra-CSF) therapy via Ommaya reservoir (OR) versus lumbar punctures (LP) may result in improved efficacy and overall survival (OS) in LC (12, 13). However, previous studies rarely touched upon subgroup analysis of patients with hydrocephalus, some uncertainty about the effect and safety remains. In this study, the purpose was to determine whether route of intra-CSF methotrexate administration influences efficacy and long-term outcome in the treatment of patients of LC with hydrocephalus.

## Methods

### Study design

This was a retrospective analysis focusing on LC patients with hydrocephalus who received intra-CSF methotrexate treatment between January 2015 and December 2021 from Department of Neurology in the Second Hospital of Hebei Medical University, Shijiazhuang, China. According to patient comfort, treatment-related complications, treatment costs and possible prognosis, the patients and their relatives select an appropriate administration route. A total of 36 patients were included. 15 patients who underwent OR placement, while 21 patients received methotrexate via repeated LP.

Diagnosis of LC was made via clinical signs and symptoms in addition to positive CSF cytology (malignant cells) and/or abnormal leptomeningeal appearance on gadolinium-contrast magnetic resonance imaging (MRI). Patients with organ failure, infectious disease of the central nervous system, systemic immune system disease, hematologic malignancy or primary brain tumor were ineligible. To increase comparability of clinical symptoms and prognosis between the two groups, this study did not include patients treated with other CSF diversion. Retrieved data regarding demographic features, date of LC diagnosis, cancer history, symptoms, Karnofsky Performance Status (KPS), type of treatment, OS, laboratory and clinical response, and complications of treatment were collected.

### Treatment plan

Within 1 week after LC diagnosis with hydrocephalus, all patients were treated with four cycles of biweekly intra-CSF methotrexate (10 mg) during the induction phase, followed by weekly for four treatments of consolidation, and then monthly maintenance therapy until disease progression (14). In addition, all patients received prophylactic concomitant dexamethasone (5 mg) to prevent chemical arachnoiditis. In case of CSF relapse, again 10 mg of methotrexate was administered according to the same treatment scheme. ICP was measured pre- and post-procedure for every patient. For the OR group, 10–15 mL CSF was quickly drained before drug infusion. To avoid cerebral herniation, LP patients received only slow, intermittent low-volume CSF drainage over an extended procedure. Though the total drained volume per LP was limited, drainage continued until post-treatment ICP normalized. Once the patients developed elevated intracranial pressure, additional CSF drainage was required by OR for symptomatic relieve. Systemic treatment, including molecular targeted agents, when administered depended on the type and state of primary cancer, and the presence of progressive systemic disease. Focal radiotherapy (RT) was indicated for palliation of LC symptoms, or for associated parenchymal brain metastasis.

### Evaluation of outcome and safety

The diagnosis date of leptomeningeal carcinoma (LC) was established as the first instance of malignant cells observed in cerebrospinal fluid (CSF) cytology or the initial MRI indicating LC. OS was defined as the interval from the date of diagnosis to the date of death from any cause. Neurological function and efficacy were assessed at baseline, after 4-week induction, and at the final visit. Consistent with prior studies (15–17), neurological status and CSF parameters were checked before each intrathecal injection for response evaluation. All clinical and cytological response assessments were based on established published criteria for leptomeningeal carcinomatosis (15–17) to minimize measurement bias. We stratified treatment response into four tiers: complete response (CR), partial response (PR), stable disease (SD), and progressive disease (PD). For patients with malignant cells detected in baseline CSF:

- CR: Full resolution of neurological symptoms and undetectable malignant CSF cells;
- PR: Improved neurological symptoms accompanied by a ≥ 50% reduction in CSF malignant cell count;
- SD: Unchanged neurological symptoms with a < 50% decline in CSF malignant cells;
- PD: New or worsening neurological deficits, or a > 25% increase in CSF malignant cells.

For patients with negative baseline CSF cytology, therapeutic response was evaluated using CSF biochemical markers:

- CR/PR: Normalized CSF glucose and protein levels alongside symptom relief;
- SD: Stable neurological symptoms with unchanged CSF biochemical parameters;
- PD: Worsened neurological symptoms and persistent abnormal CSF biochemistry.

Clinical complications and drug toxicity were assessed via physical and neurological examinations. Blood counts and biochemical markers were monitored weekly during induction and consolidation and monthly during maintenance. All patients were followed up until December 31, 2021, or until death occurred.

### Statistical analysis

Categorical data and response status between groups were compared by the chi-square test. OS curves were plotted using the Kaplan–Meier method, and intergroup survival differences were compared with the log-rank test.

All variables with univariate *p* < 0.10 were enrolled into multivariate Cox proportional hazards regression; this threshold retains clinically relevant borderline predictors. Schoenfeld residual tests confirmed the proportional hazards assumption was satisfied for all covariates. To avoid overfitting caused by limited sample size, we adopted the forced-entry model rather than stepwise selection. All screened variables plus tumor subtypes were simultaneously included for full confounding adjustment, with HRs and 95% CIs reported for each factor.

Standardized criteria for clinical and cytological response were defined in the Outcomes section to reduce measurement bias. All analyses were performed with SPSS 13.0 and R 4.0.5. Two-tailed *p* < 0.05 indicated statistical significance.

## Results

### General information

Demographic variables, clinical characteristics, MRI findings and therapeutic approaches of the patients were summarized in Table 1. Briefly, there were 19 women and 17 men with a mean age at diagnosis of 60.5 years (range 33–70), and the median KPS at LC diagnosis was 50 (range 10–90). Non-small cell lung cancer (NSCLC) (69.4%) and breast cancer (13.9%) comprised the most frequent primary cancer. The most common histology of primary cancer was adenocarcinoma (83.3%). The median time from primary cancer to LC was 24 months in the OR group, and 17 months in the LP group, respectively. LC occurred within first year of primary disease in 14 cases. Brain parenchymal metastases were present in 33% of cases. Diffuse leptomeningeal enhancement was seen in six (40.0%) cases in the OR group and seven (33.3%) cases in the LP group, while localized leptomeningeal enhancement was seen in five (33.3%) cases in the OR group and eight (38.1%) cases in the LP group. 13 patients (36.1%) had extraneural metastasis, among which bone is the most frequent target organ. Hydrocephalus was present at the same time as the LC in 12 of the 15 OR patients, and in 18 of the 21 LP patients. As for hydrocephalus types, communicating hydrocephalus was in the majority (88.8%). A total of 16 patients (44.4%) received concurrent systemic chemotherapy with negligible central nervous system penetration, and 14 patients (38.9%) received first- and second-generation EGFR-TKIs, agents with limited but detectable cerebrospinal fluid penetration that may alter leptomeningeal disease outcomes. Only a minority (19.4%) of patients received RT for LC. The leading symptoms included headache (77.8%), mental changes (25%), and visual disturbance (16.7%). With respect to the above baseline characteristics, there was no statistically significant difference between the two groups (*p* > 0 0.05).

**Table 1:** Patients characteristics.

Characteristics	OR 15 (41.2%)	LP 21 (58.8%)	Total *n* = 36	*p*
Median (range; year)	63 (46–70)	58 (33–68)	60.5 (33–70)	0.133
Sex				0.956
Female	8 (53.3%)	11 (52.4%)	19 (52.8%)	
Male	7 (46.7%)	10 (47.6%)	17 (47.2%)	
KPS, median (range)	50 (20–90)	60 (10–90)	50 (10–90)	0.628
Primary cancer types				
NSCLC	12 (80%)	13 (61.9%)	25 (69.4%)	
Breast	2 (13.3%)	3 (14.3%)	5 (13.9%)	
Stomach	1 (6.7%)	0	1 (2.8%)	
Others^a^	0	5 (23.8%)	5 (13.9%)	
Histologic type of the primary disease				
Adenocarcinoma	14 (93.3%)	16 (76.2%)	30 (83.3%)	
Non-adenocarcinoma	1 (6.7%)	2 (9.5%)	3 (8.3%)	
Lack of pathology	0	3 (14.3%)	3 (8.3%)	
Time of primary cancer to meningeal (months)	24 (0–84)	17 (0–84)	20.5 (0–84)	0.321
>12 months	10 (66.7%)	12 (57.1%)	22 (66.1%)	0.563
≤12 months	5 (33.3%)	9 (42.9%)	14 (38.9%)	
Brain parenchyma metastasis	6 (40%)	6 (28.6%)	12 (33.3%)	0.473
Leptomeningeal metastasis				0.691
Diffuse	6 (40.0%)	7 (33.3%)	13 (36.1%)	
Localized	5 (33.3%)	8 (38.1%)	13 (36.1%)	
Extraneural metastatic sites				0.319
Bone	6 (40.0%)	4 (19.0%)	10 (27.8%)	
Lung	1 (6.7%)	5 (23.8%)	6 (16.7%)	
Lymph nodes	3 (20%)	1 (4.8%)	4 (11.1%)	
Liver	1 (6.7%)	2 (9.5%)	3 (8.3%)	
Concurrent hydrocephalus at the time of LC diagnosis	12 (80%)	18 (85.7%)	30 (83.3%)	0.650
Type of hydrocephalus				0.151
Communicating hydrocephalus	12 (80.0%)	20 (95.2%)	32 (88.8%)	
Obstructive hydrocephalus	3 (20.0%)	1 (4.8%)	4 (11.2%)	
Concurrent treatment 0–2				
Targeted therapy (CNS-penetrant)	7 (46.7%)	7 (33.3%)	14 (38.9%)	
Systemic chemotherapy (limited CNS penetration)	8 (53.3%)	8 (38.1%)	16 (44.4%)	
Regional RT	3 (20%)	4 (19.0%)	7 (19.4%)	
Presenting symptoms				
Headache	12 (80%)	16 (76.2%)	28 (77.8%)	
Speech impairment	1 (6.7%)	1 (4.7%)	2 (5.6%)	
Visual disturbance	3 (20%)	3 (14.3%)	6 (16.7%)	
Sensory disturbance	1 (6.7%)	1 (4.7%)	2 (5.6%)	
Mental changes	4 (26.7%)	5 (23.8%)	9 (25%)	
Motor deficit	2 (13.3%)	3 (14.3%)	5 (13.9%)	
Seizures	3 (20%)	0	2 (5.6%)	

^a^NSCLC, non-small cell lung cancer. ^b^Other, bladder cancer and carcinoma of unknown primary.

### Comparison of response and safety

LC-related CR was reported in three (20%) and one (4.7%) patient in the OR and LP groups. PR was observed in 10 (66.7%) and nine (42.9%) patients, respectively. SD was seen in one (6.67%) patient in the OR group, and six (28.6%) patients in the LP group. PD was 6.67% in the OR group and 23.8% in the LP group. Thus, the overall clinical effective rate (CR + PR) was 86.7 and 47.6%, and there was a significant difference between the two groups (*p* = 0.016). A clinical response was observed in 100% in the OR group and 52.4% in the LP group. Negative cells were detected in three patients in the LP group, and a cytological response was obtained in 93.3% patients in the OR group and 66.7% of 18 evaluable patients in the LP group. KPS Scores ≥ 60 after treatment were observed in 13 and 12 patients, but without significant difference. Surprisingly, the use of OR was associated with decreased intracranial pressure (ICP) in all patients, compared with 16 patients via repeated LP. The difference in reducing ICP was significant (*p* = 0.042). CSF white blood cells (WBC) count of 16 patients and protein of eight patients were reduced to normal range after intra-CSF therapy, and there was no distinct difference between the two groups. In the follow-up period, complications associated with OR were recorded in two patients (13.3%), including intraventricular hemorrhage in one patient and intracranial infection in the other patient. After delayed intra-CSF administration and intravenous vancomycin respectively, the condition was nonfatal and gradually stabilized. As for adverse events associated with methotrexate, seizures caused by subacute encephalopathy occurred in two patients in the OR group, while one patient had myelosuppression and the other one developed delayed leucoencephalopathy in the LP group (Table 2).

**Table 2:** Comparison of response and safety by different administration routes.

Factor	OR (*n* = 15)	LP (*n* = 21)	*p*
Evaluation of treatment			
CR	3 (20%)	1 (4.7%)	
PR	10 (66.7%)	9 (42.9%)	
SD	1 (6.67%)	6 (28.6%)	
PD	1 (6.67%)	5 (23.8%)	
Response			0.016
Effective	13 (86.7%)	10 (47.6%)	
Ineffective	2 (13.3%)	11 (52.4%)	
Clinical response	15 (100%)	11 (52.4%)	0.002
Cytological responders	14 (93.3%)	12 (66.7%)	0.522
KPS distribution after treatment			0.058
≥60	13 (86.7%)	12 (57.1%)	
<60	2 (13.3%)	9 (42.9%)	
ICP decrease	15 (100%)	16 (76.2%)	0.042
CSF WBC down to normal	7 (46.7%)	9 (42.9%)	0.760
CSF protein down to normal	2 (13.3%)	6 (28.6%)	0.329
Complication	2 (13.3%)	0	0.085
Drug toxicity	2 (13.3%)	2 (9.5%)	0.72

### Comparison of OS

**Figure 1:**
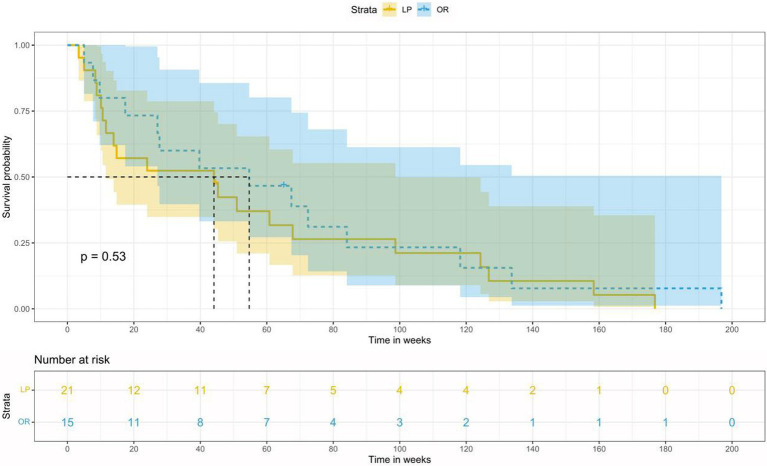
Kaplan–Meier curves relating OS. LC patients with hydrocephalus treated by OR displayed prolonged survival compared to those treated by LP. There was no statistical significance (*p* = 0.53).

**Figure 2:**
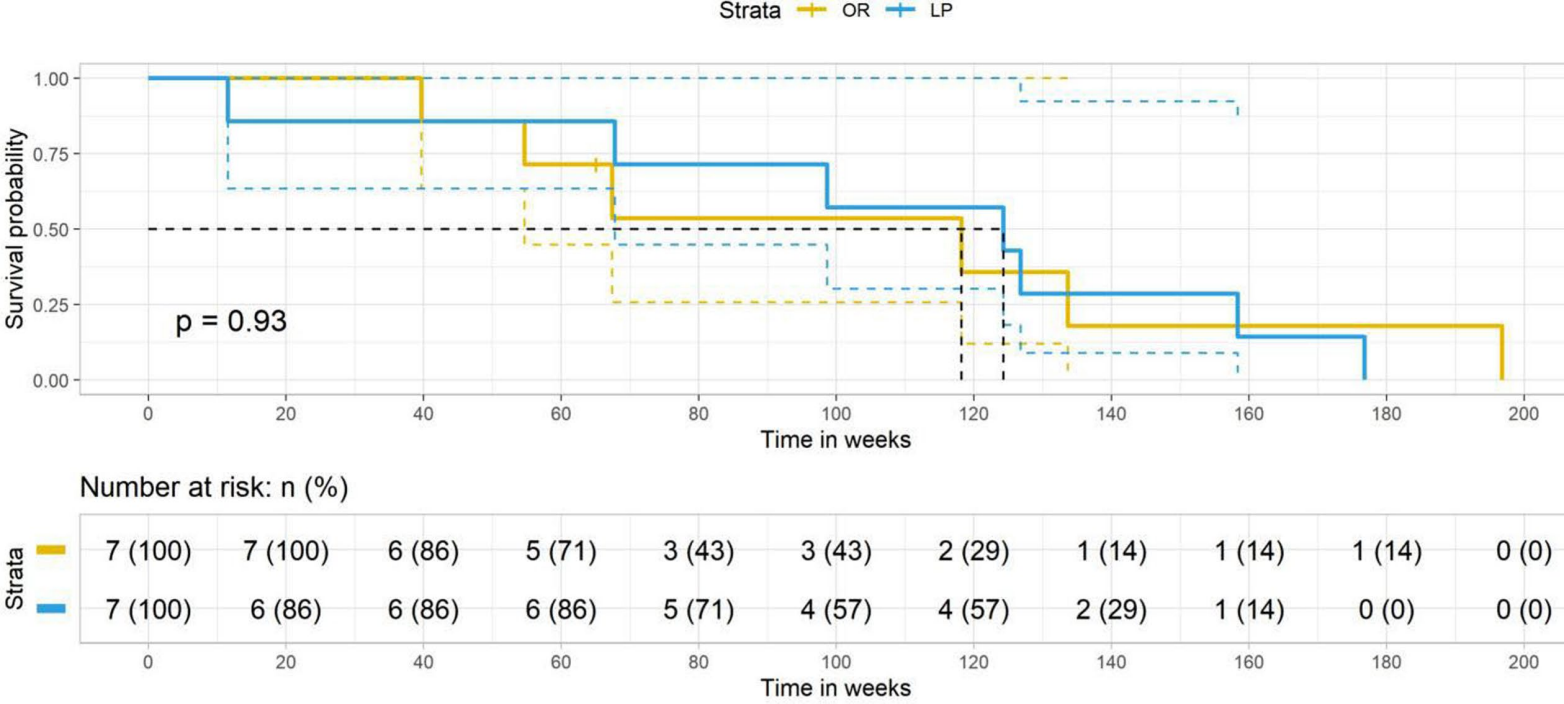
Kaplan–Meier curves relating OS. Small cell lung cancer patients with LC and hydrocephalus receiving targeted therapy displayed comparable survival between the OR and LP groups. There was no statistical significance (*p* = 0.93).

All but two patients died during follow-up. One patient in the OR group was still alive, with mild headache at the end of follow-up, 65.1 weeks after diagnosis. The other patient in the LP group was well at the end of follow-up, 44.6 weeks after diagnosis. At 1 year, estimated survival was 46.7% in the OR group and 37% in the LP group. In the OR group, the median survival period was 54.7 weeks; in the LP group, the median survival period was 44.1 weeks. No statistical difference was observed in the survival of the two groups (*p* = 0.53, Figure 1). Considering baseline heterogeneity in tumor types and imbalanced EGFR-TKI use across the full cohort, we performed stratified survival analyses restricted to NSCLC patients. Of these NSCLC patients, 7/12 in the OR group and 7/13 in the LP group received first- and second-generation EGFR-TKIs, yielding balanced targeted therapy exposure in this subset; stratified survival results are listed in Figure 2. Among EGFR-TKI-treated NSCLC patients, median OS was 96.5 weeks for OR versus 109.2 weeks for LP, with no significant intergroup difference (*p* = 0.93, Figure 2).

Table 3 lists the results of median survival by treatment and various prognostic factors. There is a trend toward longer survival in patients having the following characteristics: KPS ≥ 60 after treatment, targeted therapy, cytological responders and effective response. However, intra-CSF route, sex, age, time from diagnosis of primary to LC, primary cancer types, ICP, CSF protein and cell count after treatment did not appear to affect survival.

**Table 3:** Median survival by treatment and patients characteristics.

Factor	No. of deaths	No. of patients	Median survival (weeks)	Univariate *p*
Intra-CSF route				0.530
OR	14	15	54.7	
LP	20	21	44.1	
Sex				0.397
Female	18	19	67.4	
Male	16	17	39.7	
Age				0.324
≥60	18	20	39.7	
<60	16	16	44.1	
Time from diagnosis of primarycancer to diagnosis of LC				0.534
>12 months	21	22	39.7	
≤ 12 months	13	14	44.1	
Primary cancer types				0.551
NSCLC	24	25	45.3	
Breast	4	5	98.7	
Other	6	6	14.8	
Extraneural metastasis				0.073
Yes	13	13	11.6	
No	21	23	51	
ICP after treatment				0.478
≥300	5	5	10.6	
<300	29	31	45.3	
KPS after treatment				0.017
≥60	26	28	54.7	
<60	8	8	11.6	
CSF protein after treatment				0.450
≤45 mg/dL	15	17	27.7	
>45 mg/dL	19	19	54.7	
CSF WBC count after treatment				0.790
≤8 /mm^3^	23	24	44.1	
>8 /mm^3^	11	12	39.7	
Targeted therapy				0.000
Yes	14	15	118.2	
No	20	21	14.8	
Cytological responders				0.000
Yes	24	26	94.1	
No	7	7	11.4	
Response				0.000
Effective	21	23	72.4	
Ineffective	13	13	10.1	

**Figure 3:**
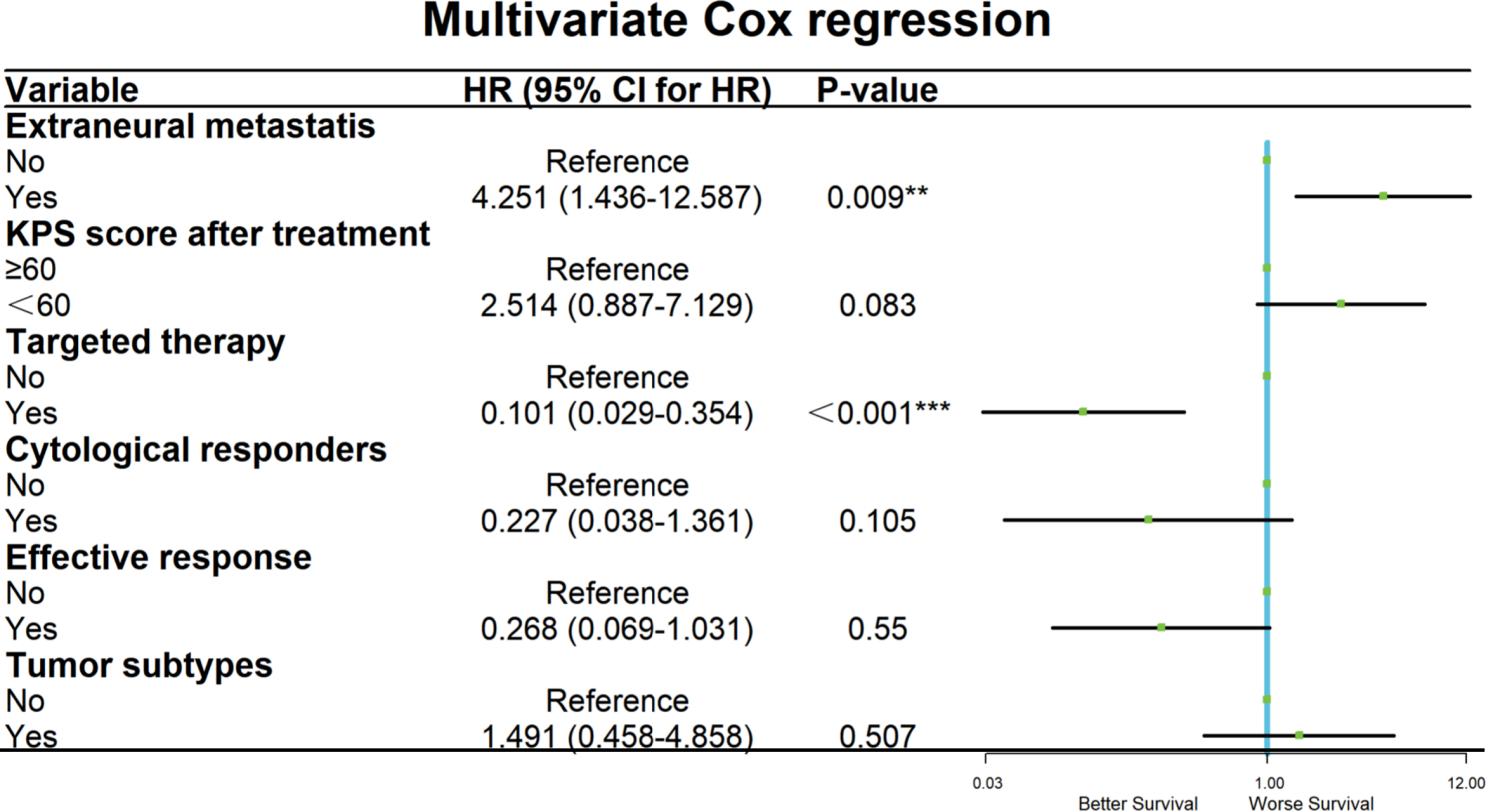
The forest plot for Multivariate Cox regression. In multivariate analysis, extraneural metastasis and targeted therapy were significantly associated with overall survival. ^*^Demonstrated that variables were significantly associated with overall survival.

Furthermore, to evaluate prognostic factors for LC, multivariate Cox regression analysis was conducted. In response to concerns regarding intergroup baseline heterogeneity, primary cancer types were additionally included in the model together with all significant variables from univariate analyses (Table 3): primary cancer types, extraneural metastasis, post-treatment KPS ≥ 60, targeted therapy, cytological response and effective treatment response (Figure 3). Two variables remained independently associated with OS: extraneural metastasis (*p* = 0.009) and targeted therapy (*p* < 0.001). Interestingly, effective response to intra-CSF chemotherapy, post-treatment KPS ≥ 60 and positive cytological responders showed prognostic value in univariate analysis, yet this association weakened after adjustment for other covariates including extraneural metastasis.

Seven patients had an unusually long survival time of more than 100 weeks. The characteristics and details regarding therapies received for LC are also summarized in Table 4. Two of them were men. Three were in the OR group. Their median age was 53.3 and median survival was 147.8 weeks. Their KPS distribution after treatment ranged from 60 to 90. Four patients had a tumor history. The great majority of patients (71.4%) had NSCLC and the pathological feature was adenocarcinoma. Extraneural metastasis was found in two patients. All of these seven patients received targeted drugs, and three patients received systemic chemotherapy and other concurrent therapy. The overall response of six patients were reported as PR, and one patient was seen as SD.

**Table 4:** Summary of the seven patients with more than 100 weeks survival.

Age Median survival (weeks)	*n* (range) 53.3 (33–69) 147.8 (118.2–196.8) *n* (%)
Sex	
Female	5
Male	2
Group	
OR	3
LP	4
KPS score after treatment	
60	2
70	2
80	2
90	1
Tumor history	4
Tumor type	
NSCLC	5
Histologic type of the primary disease	
Adenocarcinoma	5
Extraneural metastatic sites	
Bone + Lung	1
Bone + Lymph nodes	1
No	5
Other treatment	
Targeted therapy	4
Systemic chemo + Targeted therapy	2
Systemic chemo + Targeted therapy + Regional RT	1
Previous surgery	2
Evaluation of treatment	
CR	0
PR	6
SD	1

## Discussion

LC is still difficult to treat, with generally poor outcomes owing to limited therapeutic options. IT chemotherapy is the mainstay treatment for patients with LC from solid tumor and hematological malignancies, which involves the administration of drugs directly into the CSF via OR or LP, and allowing adequate distribution and long-lasting drug concentration at the target site with a low total dose and minimum systemic toxic effects, although the efficacy of intensive treatment in LC is unclear (16, 18, 19). Currently, options available to tackle hydrocephalus related to LC include ventriculoperitoneal shunt, repeated LP, OR placement and endoscopic third ventriculostomy. There is no standard therapy for a preferred management option (20). Preliminary evidence suggests OR placement offers many advantages, such as less side effects, increased convenience and comfort, more consistent drug concentration, and a high number of complete responses (21–23). This view is supported by our data showing that the effective response rate was higher in the OR group, and 13 of 15 patients responded to treatment by OR versus 10 of 21 patients treated by LP (*p* = 0.016). Meanwhile, the rate of reducing ICP was significantly higher in the OR group than the LP group (*p* = 0.042). These findings indicate that OR appropriately can relieve the clinical symptoms of LC patients more effectively. Nevertheless, the superior symptom and ICP control observed in the OR group cannot be solely attributed to intraventricular drug administration. A key procedural confounder exists between the two groups: each Osession performs rapid drainage of 10–15 mL CSF prior to chemotherapy infusion, whereas lumbar puncture only permits slow, limited-volume CSF drainage to avoid cerebral herniation, even though ICP was normalized immediately after each intervention for all participants. Repeated large-volume CSF evacuation via the Ommaya device facilitates clearance of circulating tumor cells and sustains longer-term intracranial pressure relief between treatment cycles. Given this inherent, non-standardized operational difference in our retrospective cohort, we are unable to isolate and quantify the independent therapeutic effect of the intrathecal delivery route itself.

In addition, no significant difference in cytological responders between groups was recorded (*p* = 0.522). The most reasonable explanation is that the cytology was from a lumbar or ventricular origin from start to finish, respectively. Given the same intra-CSF regimen and presence of CSF flow alteration in LC-related hydrocephalus, so an uneven distribution of the drug can partly lead to an ineffective treatment. The cytological response rates reported in other retrospective series are similar to ours. For example, a response rate was observed in 53/72 patients (74%) with leptomeningeal involvement of hematological malignancies treated with methotrexate (24). Similarly, there were also no difference in decrease of CSF WBC count and protein levels.

Undoubtedly, implantation of OR also has a series of complications, such as hemorrhage, suboptimal positioning of the ventricular catheter, proximal malfunction of the catheter and infection (25). Our data demonstrated a safety profile with no deaths nor fatal neurological deficits observed. The low infection (6.67%) and hemorrhage (6.67%) rate seen in the current series are quite similar to those reported in recent studies. Sandberg et al. (21) reported complications of OR placement occurred in 10 patients (9.3%) and included two infections, five catheter malpositions, and three intracranial hemorrhages. It was also reported that a total of 11 complications related to the use of an intraventricular access device were seen including seven infections, one intracranial hemorrhage, two instances of symptomatic leukoencephalopathy and one catheter malpositioning in 112 patients with solid cancer-related LC (23). These findings show that OR insertion appears to be safe with a low complicatin rate. In order to minimize these risks, shortening operating times, serial image examinations and use of neuronavigation application should be helpful (21, 22). At our institution, to decrease the complications, all operations are performed by a single experienced surgeon, and all intra-CSF chemotherapy administrations are performed by a single trained team using the same standardized protocol.

Methotrexate is one of most useful agents and characterised by a biphasic curve with a half-life of 8–10.5 h and a 4-day decline to sub-therapeutic levels (17). Methotrexate-induced neurotoxicity is an important clinical complication, which can be divided into acute, subacute, and chronic forms based on the time of appearance of symptoms after administration (26). Of these complications, the most common is chemical arachnoiditis, and rare types includes spinal cord lesions, seizure, and encephalopathy (26, 27). In addition, myelosuppression can also occur. The overall serious complication rate in the present study is lower than what has been previously reported with IT methotrexate alone (11% versus 21%) (15). Physicians must be aware of these complications because it is potentially reversible with early recognition and cessation of chemotherapy. Moreover, concomitant intra-CSF dexamethasone is recommended in all protocols to reduce the risk of myelosuppression and chemical meningitis.

Our study comprehensively evaluated factors prognostic for survival following aggressive multimodality therapy for LC. The median survival of patients entered onto this study were 54.7 and 44.1 weeks, respectively. In multivariable analysis one factor was noted to be significant predictor of prolong survival: targeted therapy. Conversely, OS is negatively affected by concomitant extraneural metastasis. Recently, there is increasing evidence from randomized trials concerning the benefit of concomitant targeted therapy in LC (28). A phase II prospective cohort study found an impressive intracranial response rate of 55%, a disease control rate of 77.5%, and a median OS of 16.9 months for LC from NSCLC patients treated with osimertinib (29). In general, effective response is highly prognostic for improved survival on a patient level, which has been shown in some studies (30, 31). This association was also seen in our study. Our results suggested that patients who achieved a combined cytological and neurological response may present significantly longer OS times than non-responders. Notably, no significant difference in median survival between the two groups was shown, which was inconsistent with those reported on previous clinical trials. Montes et al. (12) and Glantz et al. (13) observed significant survival advantage when comparing OR methotrexate delivery with LP delivery in adults with LC. We hypothesized that, hydrocephalus increased the likelihood of CSF flow obstruction, and further impede the reliable distribution of the chemotherapy drugs. Moreover, treatment of hydrocephalus alone could not improve OS. Similarly, one retrospective series in LC found data suggesting that surgically untreated hydrocephalus showed poor OS compared with surgically treated hydrocephalus, but without statistical significance (8). Our patients with hydrocephalus demonstrated significantly longer survival than those already available in previously reported literature (32, 33). In fact, it is difficult to compare prognosis among different studies because not only is intra-CSF treatment included, but also added RT and/or systematic chemotherapy. In addition, our candidates generally are younger patients with controlled systemic disease who otherwise have a life expectation of greater than 3 months.

There are multiple key limitations inherent to this single-center retrospective cohort study. First, the retrospective design introduces inevitable selection bias and residual confounding factors. The relatively small sample size raises risks of overfitting in multivariate Cox regression analysis and reduces the external generalizability of our results. Notably, an unstandardized procedural confounder exists between groups: OR tapping permits rapid large-volume CSF drainage before chemotherapy, while lumbar puncture only allows slow, low-volume drainage to avoid cerebral herniation. This discrepancy partially explains the intergroup gaps in sustained intracranial pressure control and cytological response, preventing us from separating the independent therapeutic effect of the intrathecal drug delivery route itself.

Second, though OR implantation relieved neurological symptoms and improved ICP control without prolonging overall survival, our observations cannot be generalized to all patients with LC and hydrocephalus. Historically, hydrocephalus is considered a relative contraindication for intra-CSF chemotherapy due to impaired CSF circulation and elevated neurotoxicity risks; we weighed risk–benefit tradeoffs for high-risk LC patients with dismal prognosis, but our findings do not constitute universal evidence to revise standard clinical guidelines. All study results require cautious interpretation. Large, prospective multicenter cohorts with unified CSF drainage protocols are necessary to further validate our preliminary conclusions.

## Conclusion

In conclusion, the findings demonstrated that compared with intra-CSF methotrexate via LP, OR administration did not prolong the OS in LC patients with hydrocephalus, but it was more effective in relieving clinical symptoms and did not increase adverse reactions. We identified that concomitant targeted therapy was shown to be favorable prognostic factors. Nevertheless, patients with extraneural metastasis had a worse outcome than those with single central nervous system involved. Our study may contribute to the assessment and management of LC with hydrocephalus in the clinic.

## Data Availability

The original contributions presented in the study are included in the article/Supplementary material, further inquiries can be directed to the corresponding author.
